# Understanding causal pathways within health systems policy evaluation through mediation analysis: an application to payment for performance (P4P) in Tanzania

**DOI:** 10.1186/s13012-016-0540-1

**Published:** 2017-02-02

**Authors:** Laura Anselmi, Peter Binyaruka, Josephine Borghi

**Affiliations:** 1 Centre for Health Economics, University of Manchester, Manchester, UK; 2 Ifakara Health Institute, Dar es Salaam, Tanzania; 3 Department of Global Health and Development, London School of Hygiene and Tropical Medicine, London, UK

**Keywords:** Policy evaluation, Pay for performance, Mediation analysis, Human resources, Health financing, Health governance, Maternal care, Tanzania

## Abstract

**Background:**

The evaluation of payment for performance (P4P) programmes has focused mainly on understanding contributions to health service coverage, without unpacking causal mechanisms. The overall aim of the paper is to test the causal pathways through which P4P schemes may (or may not) influence maternal care outcomes.

**Methods:**

We used data from an evaluation of a P4P programme in Tanzania. Data were collected from a sample of 3000 women who delivered in the 12 months prior to interview and 200 health workers at 150 health facilities from seven intervention and four comparison districts in Tanzania in January 2012 and in February 2013. We applied causal mediation analysis using a linear structural equation model to identify direct and indirect effects of P4P on institutional delivery rates and on the uptake of two doses of an antimalarial drug during pregnancy. We first ran a series of linear difference-in-difference regression models to test the effect of P4P on potential mediators, which we then included in a linear difference-in-difference model evaluating the impact of P4P on the outcome. We tested the robustness of our results to unmeasured confounding using semi-parametric methods.

**Results:**

P4P reduced the probability of women paying for delivery care (−4.5 percentage points) which mediates the total effect of P4P on institutional deliveries (by 48%) and on deliveries in a public health facility (by 78%). P4P reduced the stock-out rate for some essential drugs, specifically oxytocin (−36 percentage points), which mediated the total effect of P4P on institutional deliveries (by 22%) and deliveries in a public health facility (by 30%). P4P increased kindness at delivery (5 percentage points), which mediated the effect of P4P on institutional deliveries (by 48%) and on deliveries in a public health facility (by 49%). P4P increased the likelihood of supervision visits taking place within the last 90 days (18 percentage points), which mediated 15% of the total P4P effect on the uptake of two antimalarial doses during antenatal care (IPT2). Kindness during deliveries and the probability of paying out of pocket for delivery care were the mediators most robust to unmeasured confounding.

**Conclusions:**

The effect of P4P on institutional deliveries is mediated by financing and human resources factors, while uptake of antimalarials in pregnancy is mediated by governance factors. Further research is required to explore additional and more complex causal pathways.

## Background

### Introduction

Much of the focus of programme evaluation has been on outcome measurement and finding out whether or not a programme works, with randomised trials being considered to be the gold standard for causal inference [1]. However, when dealing with complex interventions, it is not enough to know whether they work, we also need to understand how they work [2]. Process evaluation enables us to get at the how and why questions and unpack the “black box” surrounding complex interventions and is increasingly promoted within evaluation research [3, 4].

One of the core functions of process evaluation is to shed light on causal mechanisms or the process through which a programme influences an outcome [2, 5]. Examination of causal mechanisms is necessary in order to understand why a programme worked, or why it did not work, and whether the underlying theory was sound. It enables theory building and enhances intervention design [6] and can support the plausibility of outcome effects being associated with the intervention in a non-randomised study [7], increasing the internal validity of evaluation in social sciences [1, 5].

Practically, causal mechanisms can be identified by specifying intermediate outcomes or variables, referred to as mediators, that are on the causal pathway between the intervention and the outcome [6, 8]. The approach used to investigate causal mechanisms involves the estimation of causal mediation effects or the breakdown of total causal effects into indirect effects (the effect of the intervention on the outcome that passes through the mediator) and the direct effect (the effect of the intervention on the outcome through all other pathways) [9]. Causal mediation analysis has been employed to test change pathways within the evaluation of public health programmes, using individual-level psychological [9–12] or physical characteristics [13], that may affect behaviour change outcomes. A recent study also considered the effect of community along with individual level mediators [14]. To the best of our knowledge, to date, there has been only one study [15] considering mediators which are relevant to the evaluation of interventions aimed at strengthening health systems.

Payment for performance (P4P) is an example of a programme which operates at the health system level with the aim of improving the quality and use of health services to enhance population health outcomes. P4P involves the payment of financial rewards to health workers (and sometimes to health facilities) based on their achievement of pre-specified performance targets. P4P has been widely used in the UK and the USA [16] and increasingly in low- and middle-income countries [17].

There is a growing body of evidence evaluating the impact of P4P [18]. Findings show that overall P4P has a positive effect on targeted service outcomes [19], although the evidence base in low-income settings is limited to a small number of studies [17, 20–25]. There has been less attention to the processes by which these outcomes are achieved, particularly in low- and middle-income settings [17, 26]. Three studies examined the implementation process challenges facing a P4P programme [27–29] and evaluations are increasingly looking at intermediate outcomes that may have affected service delivery [15, 30]. However, existing studies do not conclusively shed light on the pathways through which P4P achieves outcomes. Either they do not formally test the pathways or they test them on a limited number of mediators [15].

The overall aim of the paper is to test the causal pathways through which payment for performance may (or may not) influence the utilisation of maternal health services. A previous study in Tanzania evaluated the impact of P4P on service use, quality, equity, and health worker motivation over a 13-month period from January 2012 to February 2013 using linear difference-in-difference analysis [31]. The evaluation found a significant and positive effect on two of the targeted indicators: an increase of 8.2 percentage points (CI 3.6 to 12.8) in institutional deliveries, of 6.5 percentage points (CI 1.3 to 11.7) in the rate of deliveries in public facilities, and of 10.3 percentage points (CI 4.3 to 16.2) in the proportion of women receiving two antimalarial doses during antenatal care [21]. In this paper, we extend this analysis to examine the mediators of programme effect and to test the causal pathway to improved outcomes.

### Study setting

In 2011, the Ministry of Health and Social Welfare of the Republic of Tanzania introduced a P4P scheme in the Pwani region, with initial payments being made in mid-2012. The P4P scheme comprised four main components.

(1) P4P provided financial bonuses to health facilities and district and regional health managers based on achievement of maternal and child health (MCH) performance targets related to service coverage and quality of care. The targets were either for specific services (e.g., institutional delivery, postnatal care, family planning) or for care provided during a service (e.g., two doses of intermittent preventive treatment for malaria (IPT2) during antenatal care and HIV treatment for HIV-positive pregnant women). At the facility level, at least three quarters of the bonus were distributed among health workers. The health worker incentive represented about 10% of the average health worker monthly salary (about USD 30 per month). District and regional managers received bonus payments based on the performance of facilities in their district and region.

(2) The remaining 25% of the bonus went to the health facility and could be invested in drugs, supplies, or facility improvements. This represents roughly 4% of their average budget.

(3) Supervision was more frequent as facility performance data were verified every 6 months by national, regional, and district stakeholders, whereby achievements of targets, established by the Central Ministry of Health and Social Welfare, were measured and bonuses paid.

(4) Primary care facilities had to open bank accounts in order to receive bonus payments and could retain cost sharing revenue in these accounts, whereas before such funds were held at district level. Health Facility Governing Committees, comprised of health workers and community members are responsible for managing facility resources, including P4P bonus payments, and representatives were to be present to withdraw bonus funds from the bank. However, the community members on the committee were not eligible for bonus payments.

### Conceptual framework

**Fig. 1:**
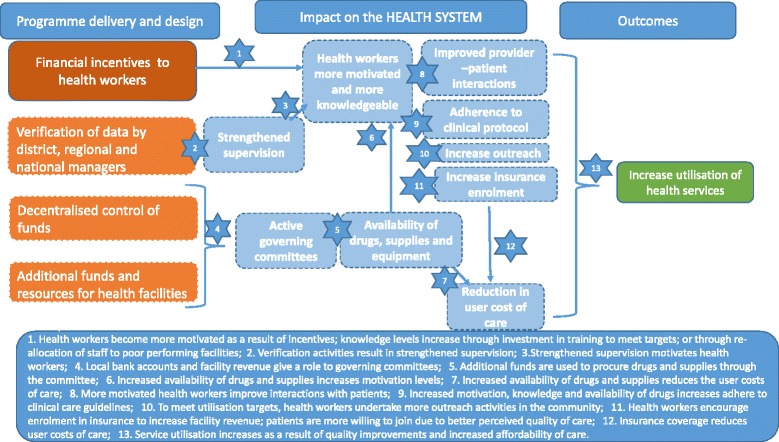
Theory of change of P4P pathways to impact via health system strengthening

Our analysis was guided by a theory of change for how P4P would affect the health system to improve outcomes and a set of underlying assumptions about the change processes involved (Fig. 1).

The increase in facility revenue from performance payments, together with financial autonomy resulting from facility-level bank accounts, may generate the need for increased accountability of resource allocation and use at the facility level, potentially stimulating health facility governing committees that are otherwise inactive and improving relations between providers and communities [32]. Greater resources and more accountability over their use are expected to lead to improved availability of equipment, drugs, and medical supplies at the facility, especially in relation to targeted services. P4P is also expected to directly affect supervision linked to the process of performance verification done by health care managers, as this results in more frequent contact between providers and managers, who examine registers and work conduct at the facility.

The direct financial incentives to health workers that are tied to service delivery, coupled with the changes in the availability of resources supervision practices are expected to impact on health workers’ job satisfaction and increase motivation to adhere to clinical guidelines [33, 34] and treat patients respectfully. Health worker knowledge may also increase, through investment in training to improve skills linked to incentivised services or through reallocation of staff to under-resourced or poor-performing facilities. To stimulate service use and achieve targets, health workers may undertake more outreach activities and/or reduce user fees and/or be more likely to enforce exemptions for vulnerable groups [35, 36] or encourage enrolment in community health insurance, as this generates additional revenue for the facility.

We identified a set of indicators to measure each of the steps on the causal pathway (Table 1). The indicators were measured the household, facility, and health worker surveys (Table 1). A full discussion of the effects of P4P on the availability of medical supplies and drugs and on governance of facilities is presented elsewhere [37].

**Table 1:** Health financing, governance and human resources indicators tested as potential mediators linked to theory of change

Steps on the causal pathway/mediators	Indicators for measurement (data source)
Strengthened supervision	- Health workers received a supervision visit in the last 90 days (health worker survey)
Active governing committees	- Health facility with a governing committee (health facility survey) - Health facility governing committees meetings held in last 90 days (health facility survey and health worker survey) - Minutes of health facility government committee meetings available (health facility survey and health worker survey) - Health workers satisfied with relationships between health facility and local leaders (health worker survey)
Availability of drugs, medical supplies and equipment	- Index of stock out of 24 essential drugs in the past 90 days (health facility survey) - Index of stock out of 5 drugs used during delivery in the past 90 days (health facility survey) - Index of stock out of medical supplies in the past 90 days (health facility survey) - Index of stock out of SP (IPTp) (health facility survey) - Index of medical equipment functionality (health facility survey)
Health worker more motivated and knowledgeable	- Intrinsic and extrinsic motivation score (health worker survey)
Improved patient provider interactions	- Kindness during delivery ranked by women on a scale of 1–10 where 1 is minimum and 10 is maximum (women survey)
Adherence to clinical protocol	- Uptake of IPT 2 during ANC (women survey)
Increased outreach activities	- Frequency of outreach visits performed by health facility staff in past 90 days (health facility survey and health worker survey) - Outreach visits conducted by health facility staff in the last 90 days (health facility survey and health worker survey)
Increase insurance enrolment	- Health insurance scheme available in the community (health facility survey) - Number of community health insurance scheme members per health facility (health facility survey)
Reduction in user charges	- Probability of payment at delivery at the health facility (or public health facility) (women survey)

Health workers kindness and knowledge have been re-ranked on a scale from 1 to 100 for ease of interpretation of the coefficients

## Methods

### Data sources

Surveys were undertaken in all seven districts in the Pwani region where P4P is being implemented and four neighbouring comparison districts with no P4P, with 75 facilities being sampled in each of the study arms, comprising 6 hospitals, 16 health centres, 11 non-public dispensaries, and 42 dispensaries. A health facility survey was conducted at all facilities and 1–2 health workers per facility were interviewed. Interviews were conducted with women who had delivered in the past 12 months sampled within the catchment area of the facilities—a total of 3000 women per round. Baseline data collection was conducted between January and March 2012 and endline data was collected 13 months later [31]. All data could be linked at the facility level [21].

### Data analysis

We used causal mediation analysis to identify steps on the causal pathway to the two significant outcomes in the main evaluation (delivery in a health facility and uptake of two doses of antimalarial drugs during pregnancy). We also considered potential mediators of a third outcome, delivery in a public health facility, as we thought that mediators may differ within public compared to non-public facilities. We assessed mediation by applying the linear structural equation model (LSEM) Baron and Kenny [6, 38]. We estimated a single-mediator model to identify the effect of P4P on mediators and the effect of the latter on institutional deliveries and coverage of antimalarials during pregnancy. We followed a four step process to assessing mediation.

#### Step1: Estimating the impact of P4P on outcomes

First, we replicated the analysis previously carried out by Binyaruka et al. [21] to evaluate the effect of P4P on the selected outcomes using a linear difference-in-difference regression model:

1 $$ {Y}_{ijt}={\beta}_0^1+{\beta}_1^1\left( P4{P}_j \times {\delta}_t\right) + {\beta}_2^1{\delta}_t+{\beta}_3^1{X}_{ijt}+{\gamma}_j+{\varepsilon}_{ijt}^1 $$

where *i* is the sample of women who gave birth in the 12 months prior to the interview in the catchment area of facility *j* at time *t. Y*_*ijt*_ is a dummy taking value 1 if the service was received by a woman and 0 otherwise. P4P_*j*_ is an indicator of whether P4P was implemented in the area where the woman was sampled from. We included facility fixed effects (*γ*_*j*_) to control for facility-level unobserved time-invariant characteristics and a dummy variable taking the value of 0 at baseline and 1 at endline (*δ*_*t*_) to account for year fixed effects. We also controlled for individual-level characteristics (education, religion, marital status, occupation, age, number of pregnancies) and household characteristics (insurance status, number of household members, household head education, and wealth based on ownership of household assets and housing particulars) that are known to affect outcomes (*X*_*ijt*_). The effect of P4P on outcomes was estimated by *β*_1_^1^. Standard errors were clustered at the health facility level.

#### Step 2: Identifying mediators

Second, we tested for the effect of P4P on each of the potential mediators identified within the theory of change (Table 1).

As in (1), we used a linear difference-in-difference regression model:

2 $$ {M}_{ijt}={\beta}_0^2+{\beta}_1^2\left( P4{P}_j \times {\delta}_t\right) + {\beta}_2^2{\delta}_t+{\beta}_3^2{X}_{ijt}+{\gamma}_j+{\varepsilon}_{ijt}^2 $$

where *M*_*ijt*_ is the potential mediator and *β*_1_^2^ indicates the effect of P4P on the mediator. All mediators were measured at the health facility level. Items collected through the health worker survey were either averaged across health workers in the same facility, when they concerned individual judgement (satisfaction and motivation), or the highest value was retained when they concerned health facility characteristics (time and content of last supervision visit). Indicators of price, satisfaction with the service received, and kindness during delivery that were measured at the individual level were averaged across women in the same facility catchment area. The woman herself was excluded from the calculation to avoid direct reverse causality and to test how the prevalent reported price and quality affected individual choice [39]. Although some mediators were measured at the individual level and some at the health facility level, Eq. 2 was estimated at the individual level for all mediators, for comparability with step 1 and step 3. Standard errors were clustered at the health facility level.

#### Step 3: Identifying direct and indirect causal effects

Third, we evaluated the effect of P4P on the outcomes of interest, by re-estimating Eq. 1, including the potential mediators *M*_*ijt*_ identified in step 2:

3 $$ {Y}_{ijt}={\beta}_0^3+{\beta}_1^3\left( P4{P}_j \times {\delta}_t\right) + {\beta}_2^3{\delta}_t+{\beta}_3^3{X}_{ijt}+{\beta}_4^3{M}_{ijt} + {\gamma}_j+{\varepsilon}_{ijt}^3 $$

We ran the analysis separately for each maternal care outcome *Y*_*i*_ and for each potential mediator *M*_*ijt*_ identified in step 2. If the estimated coefficient of *M*_*ijt*_ (*β*_4_^3^) was significant and the effect of P4P was reduced compared to that estimated in (1) (*β*_1_^3^ was smaller than *β*_1_^1^), we can infer that the effect of P4P on *Y*_*ijt*_ is mediated through *M*_*ijt*_. For each set of outcome and mediators, *β*_1_^3^ measures the direct effect of P4P on *Y*_*ijt*_ , while the mediated (or indirect) effect was calculated as the product between *β*_1_^2^ and *β*_4_^3^, and its significance verified by calculating their bootstrapped standard errors [6]. These analyses were run at the individual level. As for Eqs. 1 and 2, Eq. 3 was estimated using a linear probability model and standard errors were clustered at the health facility level.

#### Step 4: Sensitivity analysis

The identified mediators can only be considered to be “on the causal pathway” (enabling the measurement of causal mediation effects) under a set of two assumptions, referred to as “sequential ignorability”: first, the intervention assignment is independent of outcomes and mediators and, second, the observed mediator is independent of outcomes given the actual treatment status and pre-treatment confounders (there are no unmeasured confounders that affect both the mediator and the outcome) [40].

The first part of the assumption is satisfied if the treatment is assigned randomly or assumed to be random given the pre-treatment covariates [8]. The use of difference in difference regression methods allows us to control for factors that may lead to the endogenous assignment of the intervention subject to the assumption of parallel trends. We verified that the pre-intervention trends in a selection of mediators and outcomes were parallel between intervention and comparison areas [21].

The second assumption is still required to identify the causal effect of the mediator on the outcome and cannot be formally tested [8, 41]. To address this, Imai et al. [42] propose a measure of the sensitivity to unmeasured confounding. Since the level of correlation between *ε*_*ijt*_^2^ and *ε*_*ijt*_^3^ reflects the presence of unobservables affecting both the mediator and the outcome, the level at which the mediation effect would be zero provides an indication of how plausible the assumption is. The smaller the level of correlation, the less plausible the assumption. Imai et al. [42] develop their approach using a potential outcome framework and a semi-parametric approach for the identification of direct and mediated effects of the treatment. We set the prediction of potential outcomes to be based on Eqs. 1, 2, and 3 used in the LSEM, so that the sensitivity analysis would apply to the original results obtained. The sensitivity analysis provides the coefficient of correlation (rho) between *ε*_*ijt*_^2^ and *ε*_*ijt*_^3^ at which the average causal mediation effect (ACME) equals 0 [43].

The LSEM approach to mediation analysis requires no interaction between the intervention and the average causal mediation effect, in or words that the average causal mediation effect is equivalent in intervention and comparison areas. We test this assumption by introducing an interaction term between treatment and mediator in Eq. 3 and testing its significance.

Since the outcomes are observed at the individual level, but the P4P scheme is implemented at the health facility level, we test the sensitivity of our results to the level at which the analysis is carried out by re-estimating Eqs. 1 to 3 on the outcomes measured at the health facility level, based on averages of individuals within the facility catchment area.

We tested for clustering at the district level using a bootstrapping procedure which is recommended when the number of clusters is small [44, 45]. Since multiple hypothesis testing may lead to false rejection of the null hypothesis, we also applied a modified Bonferroni correction to adjust the significant threshold accounting for the correlation between the tested outcomes [30]. All statistical analyses were conducted using STATA 14.

## Results

### Descriptive statistics

The intervention and comparison groups are similar at baseline in relation to most of the outcomes and mediators considered (Table 2). However, in general, the comparison group performs slightly better than the intervention group in relation to the mediators.

**Table 2:** Summary statistics of maternal care outcomes and potential mediators at the baseline and endline and by intervention and comparison group

	Baseline							Endline						
	Intervention		Control		Total		Difference intervention control	Intervention		Control		Total		Difference intervention control
	*N*	Mean (SE)	*N*	Mean (SE)	*N*	Mean (SE)	*T* test (*P* value)	*N*	Mean (SE)	*N*	Mean (SE)	*N*	Mean (SE)	*T* Test (*P* value)
Outcome														
Facility-based delivery	1389	84.7 (36.0)	1485	86.8 (33.9)	2874	85.8 (34.9)	−2.1 (0.113)	1449	89.2 (31.0)	1462	83.1 (37.5)	2911	86.2 (34.5)	6.1 (0.000)
Public facility delivery	1389	76.7 (42.3)	1485	77.8 (41.6)	2874	77.3 (41.9)	−1.0	1449	81.6 (38.8)	1462	77.1 (42.0)	2911	79.3 (40.5)	4.5 (0.003)
Taken at least 2 doses of anti-malaria drug during pregnancy	1038	49.5 (50.0)	1212	56.7 (49.6)	2250	53.4 (49.9)	−7.2 (0.001)	1279	72.9 (44.4)	1252	69.2 (46.2)	2531	71.1 (45.3)	3.8 (0.036)
Potential mediators														
Health workers received supervision visit in the last 90 days (%)	1315	88.7 (31.6)	1489	90.8 (28.9)	2804	89.8 (30.2)	−2.1 (0.073)	1165	98.5 (12.3)	1172	84.7 (36.0)	2337	91.6 (27.8)	13.7 (0.000)
Health facility with a governing committee (%)	1202	74.3 (23.0)	1384	70.5 (45.6)	2586	72.2 (44.8)	3.8 (0.029)	1119	80.6 (39.5)	1442	74.7 (43.5)	2891	77.7 (41.7)	5.9 (0.000)
Governing committee met in the past 90 days (%)	1345	94.4 (23.0)	1444	93.0 (25.5)	2789	93.7 (24.3)	1.4 (0.124)	1395	63.4 (48.2)	1442	44.5 (49.7)	2837	53.8 (49.9)	19.0 (0.000)
Minutes of health facilities governing committee meetings available (%)	1249	92.7 (26.0)	1300	80.5 (39.7)	2549	86.5 (34.2)	12.3 (0.000)	1193	90.1 (29.9)	1077	92.9 (25.6)	2270	91.5 (28.0)	−2.8 (0.016)
Health worker satisfied with relationship with local leaders (%)	1374	63.2 (48.3)	1489	58.2 (49.3)	2863	60.6 (48.9)	4.9 (0.000)	1430	63.1 (48.3)	1462	57.1 (49.5)	2892	60.1 (49.0)	6.0 (0.001)
Drug stock-out index-general (0–1 index) (%)	1323	54.6 (23.3)	1468	45.7 (27.8)	2791	49.9 (26.2)	8.9 (0.000)	1449	37.6 (25.3)	1462	45.7 (29.7)	2911	41.7 (27.8)	−8.0 (0.000)
Drugs at delivery-stock-out index (0–1 index) (%)	1323	54.3 (31.1)	1448	38.6 (34.4)	2771	46.1 (33.8)	15.7 (0.000)	1449	36.7 (32.6)	1462	48.8 (37.9)	2911	42.8 (35.9)	−12.1 (0.000)
Oxytocin injection stock-out last 90 days (%)	1302	42.0 (49.4)	1448	17.3 (37.9)	2750	29.0 (45.4)	24.7 (0.000)	1449	22.3 (41.6)	1462	33.7 (47.3)	2911	28.0 (44.9)	−11.4 (0.000)
Ergometrin injection stock-out last 90 days (%)	1286	65.4 (47.6)	1408	51.3 (50.0)	2694	58.1 (49.4)	14 (0.000)	1449	42.9 (49.5)	1462	57.1 (49.5)	2911	50.1 (50.0)	−14.2 (0.000)
Misoprostol tablet stock-out last 90 days (%)	1302	58.8 (49.2)	1428	49.1 (50.0)	2730	53.7 (49.9)	9.7 (0.000)	1449	44.8 (49.7)	1462	55.7 (49.7)	2911	50.3 (50.0)	−10.9 (0.000)
Medical supplies stock-out index (0–1 index) (%)	1180	39.3 (25.3)	1227	25.6 (23.2)	2407	32.3 (25.2)	13.8 (0.000)	1449	20.0 (19.1)	1462	21.3 (18.4)	2911	20.6 (18.8)	−1.3 (0.060)
Index of stock out of IPTp last 90 days (%)	1302	27.3 (44.6)	1486	19.5 (39.5)	2770	23.1 (42.1)	7.9 (0.000)	1449	6.8 (25.1)	1462	10.9 (31.2)	2911	8.9 (28.4)	−4.2 (0.000)
Service delivery disrupted due to broken equipment last 90 days (%)	1323	24.8 (43.2)	1468	12.5 (33.0)	2791	18.3 (38.7)	12.3 (0.000)	1392	5.2 (22.2)	1462	6.4 (24.4)	2854	5.8 (23.3)	−1.2 (0.174)
Intrinsic motivation score	1374	−7.7 (95.7)	1489	7.7 (74.1)	2863	0.3 (85.4)	−15.4 (0.000)	1429	−7.2 (93.2)	1462	8.05 (73.7)	2891	0.5 (84.3)	−15.3 (0.000)
Extrinsic motivation score	1374	−13.7 (68.2)	1489	1.1 (82.0)	2863	−6.0 (76.1)	−14.8 (0.000)	1430	−12.0 (68.5)	1462	0.5 (80.4)	2892	−5.7 (75.0)	−12.6 (0.000)
Health worker knowledge (%) ^a^	1299	47.0 (27.4)	1467	67.7 (23.2)	2766	57.9 (27.0)	−20.0 (0.000)	1388	57.7 (9.8)	1347	59.6 (8.7)	2735	58.6 (9.3)	−19 (0.000)
Kindness at delivery (%) ^a,b ^	1389	74.8 (10.9)	1485	79.0 (10.1)	2874	77.0 (10.7)	−4.2 (0.000)	1449	88.9 (9.1)	1452	83.7 (11.7)	2911	83.8 (10.5)	0.21 (0.590)
Outreach visits conducted by health facility staff in the last 90 days (%)	1358	60.6 (48.9)	1424	58.5 (49.3)	2782	59.5 (49.1)	2.1 (0.258)	1449	73.5 (44.1)	1462	60.0 (49.0)	2911	66.7 (47.1)	13.4 (0.000)
Frequency of outreach visits performed by staff in last 90 days	1358	2.0 (2.8)	1424	2.0 (2.2)	2782	2.1 (2.5)	−0.0 (0.843)	1449	2.5 (3.27)	1462	2.03 (3.0)	2911	2.3 (3.1)	0.48 (0.000)
Health insurance scheme available in the community (%)	1358	79.8 (40.1)	1429	55.9 (49.7)	2787	67.5 (46.8)	24.0 (0.000)	1449	84.0 (36.7)	1462	62.4 (48.4)	2911	73.2 (44.3)	21.5 (0.000)
Number of community health insurance scheme members per HF	991	23.3 (40.9)	1144	14.8 (31.9)	2135	18.8 (36.6)	8.5 (0.000)	1369	36.9 (52.9)	1424	32.5 (56.2)	2793	34.7 (54.6)	4.3 (0.038)
Percentage of women who paid for delivery in a HF (%)^b^	1389	19.9 (20.0)	1485	14.3 (23.8)	2874	17.0 (22.2)	5.5 (0.000)	1449	0.133 (0.205)	1.462	0.119 (0.222)	2911	0.126 (0.214)	0.014 (0.082)

^a^ Kindness and knowledge originally ranked in from 1 to 10, but rescaled here from 1 to 100 for ease of interpretation of the coefficient

^b^ Calculated for each woman as average of the responses of all women in the same health facility catchment area, excluding the woman herself

### Mediation analysis

As it has been previously reported, there was a positive and significant effect of P4P on the rate of institutional deliveries (an 8.2 percentage point increase, CI 3.6 to 12.8), on the rate of deliveries in public health facilities (a 6.5 percentage point increase, CI 1.3; 11.7) and on the uptake of two doses of antimalarial drugs during antenatal care (a 10.3 percentage point increase, CI 4.4; 16.1) [21] (Table 3). The effect of P4P was tested on all potential mediators in Table 2, but results are reported only for those significantly affected by P4P (Table 3).

**Table 3:** Effect of P4P on institutional delivery and on potential mediators

	Effect of P4P (% change)	Observations
Maternal care outcomes		
Facility-based delivery	8.2*** (3.6; 12.8)	5747
Public facility delivery	6.5** (1.3; 11.7)	5747
Taken at least 2 doses of anti-malaria drug	10.3*** (4.4; 16.1)	4759
Potential mediators		
Health workers received supervision visit in the last 90 days	18.0** (4.0; 32.0)	5100
Drug stock-out index-general (0–1 index)	−17.2***^BS^ (−28.6;−5.8)	5659
Drugs at delivery stock-out index (0–1 index)	−27.0***^BS^ (−43.4;−10.6)	5639
Oxytocin injection stock-out last 90 days	−36.2***^BS^ (−55.9;−16.4)	5618
Ergomentrin injection stock-out last 90 days	−26.1** (−48.2;−4.0)	5562
Medical supplies stock-out index (0–1 index)	−14.8***^BS^ (−24.8;−4.9)	5278
Service delivery disrupted due to broken equipment last 90 days	−14.9** (−29.3;−0.4)	5602
Health worker knowledge	18.8***^BS^ (10.4; 27.2)	5461
Kindness ranks for health workers at delivery	4.3* (−0.4; 9.0)	5747
Percentage of women who paid for delivery in a HF	−4.5* (−9.5; 0.6)	5750

*BS* Significant at 5% level with Bonferroni adjusted *p* value for multiple outcomes: Bonferroni adjusted *p* value for potential mediators within three major groups: Financing 0.0047, governance 0.0017, human resources 0.0414. Indicators within each category in Table 1 have been grouped into: governance (strengthened supervision, active governing committees, increased outreach activities), financing (availability of drugs, medical supplies and equipment, increase insurance enrolment, reduction in user charges), and human resources (health worker more motivated and knowledgeable, improved patient provider interactions, adherence to clinical protocol). 95% confidence intervals in parentheses. Percent sign (%) indicates percentage points change

*p < 0.10; **p < 0.05; ***p < 0.01

P4P led to an increased availability of resources at the facility, notably a reduction in the disruption of services due to broken equipment (by −14.9 percentage points, CI −29.3 to −0.4); a reduction in the stock-out rate of essential medical supplies (by −14.8 percentage points, CI −24.8 to −4.9) and drugs (by −17.2 percentage points, CI −26.8 to −5.8), particularly those used during delivery including Oxytocin (by −36.2 percentage points, CI −55.9 to −16.4) and Ergometrin (by −26.1 percentage points, CI −48.2 to −4.0). P4P resulted in more frequent supervision. There was an increase in the probability of having received the last district or regional supervision in the last 90 days (by 18 percentage points, CI 4.0 to 32.0). P4P resulted in a significant increase in health worker knowledge (by 18.8 percentage points, CI 10.4 to 27.2) and improved patient-provider interactions, measured by patient perceptions of provider kindness during deliveries (by 4.3 percentage points, CI −0.4 to 9.0). P4P led to a reduction in user costs (by 4.5 percentage points, CI -9.5 to 0.6), measured as the reduced probability of paying out-of-pocket for institutional delivery by women living within the catchment area of the facility (Table 3). No effect was found on the remaining indicators on the causal pathway, notably, health worker motivation, outreach activities, and insurance enrolment.

Among all the potential mediators identified, only a limited number of them significantly mediated the effect of P4P on the outcomes of interest (Table 4). The coefficient associated with P4P reported in Table 4 represents the direct programme effect when controlling for a given mediator; where this is less than that reported in the analysis without mediators, there is evidence of mediation. The indirect effect of P4P on the outcome, or the effect which passes through a given mediator, is calculated by interacting the coefficient associated with the mediator of interest in Eq. 3 with the effect of P4P on the same mediator in Eq. 2. The estimates of the direct and indirect (through the selected mediators) effects of P4P on outcomes are reported in Table 5 along with the results of sensitivity to the sequential ignorability assumption (rho at which ACME equals 0).

**Table 4:** Effect of P4P and potential mediators on maternal care outcomes (results from Eq. 3)

	Facility-based delivery (% change)	Public facility delivery (% change)	Taken at least 2 doses of anti-malaria drug (% change)
**Effect of P4P without mediators**	8.2*** (3.6; 12.8)	6.5** (1.3; 11.7)	10.3*** (4.4; 16.1)
**Effect of P4P mediated by**			
**Health worker received supervision in the last 90 days**			
** - Coefficient on P4P (B**_**1**_ **)**	5.6** (4.0; 10.7)	4.3 (−1.5; 10.2)	10.7*** (4.1; 17.3)
** - Coefficient on mediator (B**_**4**_ **)**	3.1 (-2.3; 8.5)	0.8 (-6.6; 8.2)	8.5*** (2.4; 14.6)
Drug stock-out index-general (0-1 index)			
- Coefficient on P4P (B_1_)	7.8*** (3.0; 12.6)	5.5** (0.2; 10.9	11.5*** (5.5; 17.4)
- Coefficient on mediator (B_4_)	−3.6 (−9.2; 1.9)	−2.9 (−9.0; 3.2)	7.0 (−1.8; 15.8)
Drugs at delivery -stock-out index (0–1 index)			
- Coefficient on P4P (B_1_)	7.4*** (2.5; 12.3)	4.6* (−0.6; 9.9)	10.6*** (4.6; 16.5)
- Coefficient on mediator (B_4_)	−3.2 (−7.6; 1.2)	−4.3* (−9.0; 0.3)	0.8 (−5.6; 7.2)
**Oxytocin injection stock-out last 90 days**			
** - Coefficient on P4P (B**_**1**_ **)**	6.3*** (1.5; 11.1)	3.8 (−1.6; 9.1)	10.7*** (4.6; 16.9)
** - Coefficient on mediator (B**_**4**_ **)**	−4.9*** (−8.4;−1.4)	−5.3*** (−8.9;−1.7)	0.9 (−4.2; 6.1)
Ergometrin injection stock-out last 90 days			
- Coefficient on P4P (B_1_)	8.1*** (2.8; 13.4)	5.7** (0.1; 11.4)	10.8*** (4.7; 16.8)
- Coefficient on mediator (B_4_)	1.0 (−3.7; 5.6)	0.5 (−4.4; 5.5)	0.7 (−4.0; 5.4)
Medical supplies stock-out index (0–1 index)			
- Coefficient on P4P (B_1_)	8.0*** (3.1; 12.9)	4.9* (−0.8; 10.6)	10.9*** (4.3; 17.5)
- Coefficient on mediator (B_4_)	−6.8 (−16.5; 2.8)	−8.0 (−18.2; 2.2)	1.3 (−9.8; 12.4)
Service delivery disrupted due to broken equipment last 90 days			
- Coefficient on P4P (B_1_)	7.7*** (3.0; 12.4)	5.4** (0.1; 10.6)	9.9*** (3.8; 16.0)
- Coefficient on mediator (B_4_)	−3.7 (−8.8; 1.5)	−3.7 (−10.3; 2.9)	−0.5 (−8.8; 7.7)
Health worker knowledge			
- Coefficient on P4P (B_1_)	8.6*** (4.0; 13.2)	6.6** (1.5; 11.7;)	11.2*** (4.7; 17.6)
- Coefficient on mediator (B_4_)	−5.6 (−14.5; 3.2)	−1.7 (−12.0; 8.7)	−3.4 (−17.1; 10.3)
**Health worker kindness at delivery**			
** - Coefficient on P4P (B**_**1**_ **)**	4.3 (−2.2; 10.8)	3.3 (−3.2; 9.9)	10.0*** (4.0; 15.9)
** - Coefficient on mediator (B**_**4**_ **)**	9.1*** (6.4; 11.8)	8.0*** (4.7; 10.3)	0.3 (−1.9; 26)
**Percentage of women who paid for delivery in a HF**			
** - Coefficient on P4P (B**_**1**_ **)**	4.0 (−2.5; 10.5)	1.5 (−5.0; 8.0)	9.4*** (3.5; 15.3)
** - Coefficient on mediator (B**_**4**_ **)**	−93.5*** (−122.7;−64.4)	−112.9*** (−141.0;−84.8)	−17.9* (−35.8; 0.1)
Taken at least 2 doses of anti-malaria drug during pregnancy			
- Coefficient on P4P (B_1_)	9.3*** (2.5)	8.2*** (2.9)	
- Coefficient on mediator (B_4_)	1.4 (1.2)	2.1* (1.2)	

95% confidence intervals in parentheses; percent sign (%) indicates percentage points change; bold font indicates identified mediators

*p < 0.10; **p < 0.05; ***p < 0.01

**Table 5:** Indirect effect of potential mediators on maternal care outcomes

	Facility based delivery (% change)	Public facility delivery (% change)	Taken at least 2 doses of anti-malaria drug (% change)
P4P			
Total effect	8.2***	6.5***	10.3***
Standard error	(3.6; 12.8)	(1.3; 11.7)	(4.4; 16.1)
Indirect effect of P4P on outcome through mediators			
Health worker received supervision in last 90 days			
Indirect effect (coefficient interaction)			1.5***
Confidence Interval			(0.1, 3.0)
Percentage of total effect explained			15
Correlation at which ACME = 0			−0.0343
Oxytocin injection stock-out			
Indirect effect (coefficient interaction)	1.8***	1.9***	
Confidence Interval	(0.7, 2.9)	(0.8, 3.1)	
Percentage of total effect explained	22	30	
Correlation at which ACME = 0	−0.0439	−0.0437	
Average kindness (excluding woman herself)			
Indirect effect (coefficient interaction)	3.9***	3.2***	
Confidence interval	(3.0, 4.7)	(2.4, 3.9)	
Percentage of total effect explained	48	49	
Correlation at which ACME = 0	0.2085	−0.0202	
Mean of women who paid for delivery (excluding woman herself)			
Indirect effect (coefficient interaction)	3.9***	5.0***	
Confidence interval	(3.0, 4.8)	(3.9, 6.3)	
Percentage of total effect explained	48	78	
Correlation at which ACME = 0	−0.2326	−0.2590	

Results obtained from LSEM model. Correlation at which ACME = 0 is derived using Imai et al. sensitivity analysis. ﻿*p﻿ < 0.10; **p < 0.05; ***p < 0.01

The probability of paying for delivery and the perceived kindness of health workers during delivery mediate the effect of P4P on institutional deliveries, and the stock out rate of Oxytocin mediates the effect of P4P on deliveries in public facilities. When these are included as mediators, P4P has no significant direct effect on the outcome (Table 4).

The reduction in the proportion of women who paid for delivery mediates 48% of the effect of P4P on institutional delivery and 78% of the effect of P4P on delivery in a public health facility (Table 5). The reduction in the stock-out rate of oxytocin mediates 22% of the total effect of P4P on institutional delivery and 30% of the total programme effect on delivery in a public health facility (Table 5, columns 1 and 2). The kindness of providers during delivery mediates 48% of the total effect of P4P on institutional deliveries and 49% on deliveries in public facilities. The increase in the timeliness of supervision mediates 15% of the effect of P4P on the uptake of two doses of anti-malarial drugs during antenatal care (Table 5, column 3), but did not mediate the effect of P4P on institutional deliveries. Uptake of two doses of anti-malarial drugs did not appear to be a significant mediator of the effect of P4P on institutional deliveries (Table 4, columns 1 and 2), but it was borderline significant for deliveries in a public health facility.

### Sensitivity analysis

The sensitivity analysis (Table 5 and Table 9 in the Appendix) indicates that little correlation between the error terms of Eqs. 2 and 3 (correlation coefficients ranging from 0.02 to 0.04) would be sufficient to reduce the mediated effect to zero for most mediators. However, a higher correlation coefficient would be required to reduce to zero the indirect effect of P4P through a reduction of payment at delivery and increased health worker kindness, on institutional delivery (correlation coefficients 0.23 and 0.20, respectively) and on delivery in a public health facility (correlation coefficients 0.25 and 0.16, respectively).

When carrying out the analysis at the health facility level (Table 6, Table 7 and Table 8 in Appendix), the stock out rate of Oxytocin and the perceived kindness of health workers at delivery still mediated the effect of P4P on institutional deliveries, while the proportion of women who paid for delivery mediated the effect on deliveries in public facilities. However, the other mediators identified were no longer significant and no mediators for the uptake of two doses of anti-malarial drugs during antenatal care were identified. New mediators were also identified. For example, health worker satisfaction with local leaders became mediator of delivery in a public health facility. None of the indirect effects were significant, however, as a consequence of the reduced statistical power due to the smaller number of observations.

A number of other sensitivity analyses were carried out. We tested for significance of the interaction between treatment and mediator in Eq. 3 and found no significant effect indicating that the average mediation effect is equivalent in treated and non-treated areas. We identified the same set of potential mediators when we tested for the effect of P4P correcting standard errors for clustering at the district level. When we adjusted the level of significance to account for multiple outcome testing, the reduction in the stock out rate of Oxytocin was the only mediator that remained significant.

## Discussion

Causal mediation analysis has been put forward as an approach to understand causal mechanisms within process evaluation [2]. However, to date, there is very little empirical evidence of its application within the evaluation of complex health interventions. Building on an existing impact evaluation, we set out to test the causal pathways through which P4P affected maternal care outcomes using causal mediation analysis. While our finding of P4P effects on core maternal outcomes is partly consistent with previous evaluation studies in Rwanda and Burundi [20, 22, 30, 46], ours is the first to formally test the pathways through which P4P affects outcomes.

As in a previous study [15], we found that P4P affects the level of inputs available in health facilities. However, we tested for a wider range of mediators consistently with our theory of change and found that they mediate a significant proportion of the effect of P4P on the use of maternal care services.

Reductions in the probability of paying out of pocket and increased provider kindness during delivery mediated the largest share of the P4P effect on institutional deliveries overall and in public facilities, and these mediation effects were more robust to unmeasured confounding. Oxytocin is a drug administered to induce or support labour and to manage the third stage of labour reducing the risk of postpartum haemorrhage [47]. The reduction in the rate of stock-out of Oxytocin mediated 22% of the effect on institutional delivery (up to 30% in public health facilities), but the correlation coefficient at which the ACME is zero was very low (0.04) suggesting that the results are highly sensitive to unmeasured confounding. The effect of P4P on the availability of Oxytocin is, however, consistent with our theory of change. The increased availability of Oxytocin may be due to additional resources made available through P4P to facilities and/or greater communication with district authorities resulting from more frequent supervision. The increased availability of Oxytocin may be appreciated by women as a marker for quality of obstetric care, and management of bleeding, thereby influencing demand [48], though there is no literature highlighting women’s preference for induction [49].

Although women are supposed to be exempt from payment for deliveries in public facilities, often such exemptions are incompletely enforced [50]. Also, when drugs are out of stock, women have to pay for them at private pharmacies. The mediation effect of the probability of paying for care is consistent with providers making a concerted effort to enforce exemptions to attract women to facilities for their delivery [35]. The probability of payment is also likely affected by the reduction in stock out of drugs related to delivery that no longer have to be paid for privately by patients.

Health worker kindness, measured as the mean rank reported by other women in the same health facility catchment area, was found to be a significant mediator, suggesting that increased institutional deliveries could be due to expectations of higher quality of the service provided. This is consistent with our theory of change, whereby health workers modify their interactions and behaviour with patients to make services more attractive, to increase demand so as to meet the performance targets. Literature from a range of settings has highlighted the importance of provider attitude and kindness for women’s demand for care at birth [51, 52]. Improved timeliness of supervision, which we believe may be associated with the verification activities carried out as part of the P4P programme, significantly mediated 15% of the effect of P4P on the uptake of two doses of antimalarials during pregnancy. This indicates that increased monitoring and coaching may lead health workers to improve service delivery.

Referring back to our initial theory of change, the mediators which explained the largest share of total programme effect, and were most robust to unmeasured confounding, rely primarily on health worker response to the direct financial incentive. However, we did not find evidence of P4P increasing motivation, which was identified as a necessary precursor to behaviour change within the theory of change. This could be due to the limited sample size for the health worker survey, or invalid measurement of the underlying motivation construct, which was proxied as job satisfaction. It is also possible that health workers respond to incentives by changing their behaviour without experiencing greater job satisfaction. Our results also suggest that other components of the P4P programme were relevant to outcome achievements, notably the additional availability of resources used to procure drugs and supplies, and more timely supervision, though these effects were less robust to unmeasured confounding. We found less evidence of the effect of the increased facility financial autonomy. Ultimately, such information is useful as it helps identify the programme’s most effective components and “levers” of change.

In addition to identifying likely mediators on the pathway to outcomes, our analysis also illustrates the application of causal mediation analysis to the evaluation of a health systems intervention, such as P4P, and specifically the consideration of health systems mediators, rather than individual level mediators, related to behaviour change. However, doing so does raise practical challenges.

First, when mediators operate at the level of the provider or health facility and outcomes are measured at the household or individual level, it is unclear at which level the analysis should be carried out. We carried out the analysis at the individual level, as we were interested in the pathways to population outcomes, but we assessed the robustness of results to analysis at the facility level, and we found this did affect some of the mediators. The difference in results is in part due to the weighting based on the relative size of the  health facility catchment population, which varies from facility to facility, as well as the reduced sample size and resulting lower statistical power.

Second, randomised trials of health systems interventions are often difficult to implement, and quasi-experimental methods may be the only way to assess causal effects, as in this study. However, to date, causal mediation analysis has only been used alongside randomised controlled trials. We demonstrated its use within difference-in-difference analysis. This approach rests on the assumption of parallel trends between intervention and comparison groups in relation to outcomes as well as mediators. While we were able to assess pre-intervention trends in outcomes, we could do it for only some mediators [21, 37]. In the future, researchers should seek to gather pre-intervention time series data on outcomes as well as mediators. As in the main impact evaluation [21], we used a linear regression model to estimate P4P effects which allows us to use linear structural equation modelling to generate our estimate of mediation effect, although our outcomes and many of our mediators are binary. We had, however, previously demonstrated the robustness of our results to the use of non-linear models [21].

The selection of mediators for inclusion in the analysis was limited to those available within the surveys, so that the effect through potentially relevant mediators, such as the level of funding available at the facility, could not not be tested. Our approach relies on the accurate measurement of potential mediators and, where possible, we used tools that had been tested and applied in previous research to minimise the risk of bias. Future studies should consider using qualitative methods to validate and help explain mediators identified as being significant through mediation analysis.

The application of causal mediation analysis to the evaluation of P4P generates an estimate of average causal pathways. The assumption is that all facilities experience the same pathway to impact; however, it is of course possible that facilities introduce different strategies to achieve outcomes and that there is some variation in pathways across facilities.

The assumption that interventions affect mediators, which in turn affect outcomes, presupposes a temporal ordering, of the change in mediators preceding that of outcomes. In our study, we measured outcomes and mediators at two points in time: at baseline and endline. Hence, changes in mediators were measured at the same time as changes in outcomes. In the case of mediators measured at the individual level, this was problematic, as we would not expect a woman’s report of kindness during her delivery to affect her delivery choice, rather we would expect her choice to be based on perceptions of kindness from the experience of other women. For this reason, we estimated the mediator excluding the woman herself. Further studies should seek to obtain measures of the mediator prior to that of outcomes, either through midline surveys or by framing questions appropriately (for example, did you perceive that kindness during delivery had improved at your nearby facility prior to your birth?).

While we were able to identify significant mediators and explain how much of the overall effect of P4P each could explain, we were unable to determine the order of the causal chain. Some mediators may cause other mediators; hence, there is likely to be a hierarchy of outcomes (for example, increased availability of Oxytocin may affect health worker kindness, as increased drug availability improves their ability to do their job, which in turn affects service uptake). Epidemiology offers methods for quantifying the effects of multiple mediators, and their interactions, and decomposing them, but these methods are still very recent and with limited application [10, 11, 13, 41]. Most importantly, they rely on identifying assumptions, which are often unlikely to be satisfied or hard to prove within policy experiments. Further analysis should explore ways to examine more complex causal pathways, for example, interactions between financing and human resources or governance factors, and to assess total mediated effect.

## Conclusions

In this study, we found that the effect of P4P on institutional deliveries was mediated by a reduction in the probability of women paying for delivery care and an increase in provider kindness during deliveries and greater availability of drugs. The increase in coverage of IPT during antenatal care was mediated by more frequent supervision visits.

This study illustrates that there is great potential to apply the method of causal mediation analysis to help unpack the causal mechanisms of complex health systems interventions such as P4P, shedding light on how they impact the health system to achieve population health goals. We encourage further research of this kind to strengthen the evidence base about how health system interventions works.

## Appendix

### Sensitivity analysis with data averaged at the health facility level

**Table 6:** Effect of P4P on institutional delivery and on potential mediators

	Effect of P4P (% change)	Observations
Maternal care outcomes		
Facility based delivery	7.87*** (2.4, 13.3)	300
Public facility delivery	7.4*** (0.9; 13.9)	300
Taken at least 2 doses of anti-malaria drug	11.2*** (3.9, 18.5)	300
Potential mediators		
Health worker received supervision in last 90 days	19.2** (0.2; 38.1)	266
Governing committee met in the past 90 days	30.1*** (8.1; 52.1)	291
Drugs at delivery-stock-out index (0–1 index)	−31.3*** (−51.8;−10.8)	294
Oxytocin injection stock-out last 90 days	−38.2*** (−62.8;−13.5)	293
Ergometrine injection stock-out last 90 days	−34.9** (−62.7;−7.0)	290
Antimalarial drugs stock-out last 90 days	−22.8** (−42.6;−3.0)	294
Service delivery disrupted due to broken equipment in last 90 days	−18.6** (−36.5;−0.7)	292
Health worker knowledge	18.9*** (8.4; 29.4)	300
Health worker kindness at delivery	3.27 (−2.42; 9.0)	300
Percentage of women who paid for delivery in a HF	−7.6** (-13.6; -1.6)	300

95% confidence intervals in parentheses; percent sign (%) indicates percentage points change

**p* < 0.10; ***p* < 0.05; ****p* < 0.01

**Table 7:** Effect of P4P and potential mediators on maternal care outcomes (results from Eq. 3)

	Facility-based delivery (% change)	Public facility delivery—public health facilities only (% change)	Taken at least 2 doses of anti-malaria drug (% change)
**Effect of P4P without mediators**	7.9***	7.4**	11.2***
	(2.4; 13.3)	(0.9; 13.9)	(3.9, 18.5)
**Effect of P4P mediated by**			
Health worker received supervision in last 90 days			
- Coefficient on P4P (B_1_)	3.7	0.3	12.8***
	(−3.1; 10.4)	(−7.4; 8.1)	(4.5; 21.2)
- Coefficient on mediator (B_4_)	0.3	-2.7	8.7*
	(-6.9; 7.6)	(-11.0; 5.6)	(-0.2; 17.6)
Governing committee met in the past 90 days			
- Coefficient on P4P (B_1_)	8.0***	5.7*	11.8***
	(2.2; 13.8)	(−0.9; 12.3)	(3.9; 19.8)
- Coefficient on mediator (B_4_)	1.8	−1.4	1.0
	(−2.9; 6.5)	(−6.7; 4.0)	(−5.5; 7.4)
Drugs at delivery-stock-out index (0–1 index)			
- Coefficient on P4P (B_1_)	7.1**	3.0	11.5***
	(1.4; 12.9)	(−3.5; 9.5)	(3.6; 19.4)
- Coefficient on mediator (B_4_)	−4.2*	−5.5*	−1.2
	(−9.2; 0.7)	(−11.0; 0.1)	(−8.0; 5.5)
**Oxytocin injection stock-out last 90 days**			
**- Coefficient on P4P (B**_**1**_ **)**	5.8**	1.9	12.3***
	(0.2; 11.5)	(−4.5; 8.3)	(4.3; 20.3)
**- Coefficient on mediator (B**_**4**_ **)**	−6.3***	−6.9***	0.4
	(−10.3; 2.3)	(−11.5;−2.3)	(−5.2; 6.1)
Ergometrin injection stock-out last 90 days			
- Coefficient on P4P (B_1_)	8.3***	4.5	11.9***
	(2.3; 14.3)	(−2.2; 11.2)	(3.8; 20.0)
- Coefficient on mediator (B_4_)	0.6	0.2	−0.9
	(-3.2; 4.5)	(-4.2; 4.5)	(-6.1; 4.3)
Antimalarials stock-out last 90 days			
- Coefficient on P4P (B_1_)	7.9***	5.7*	13.0***
	(2.9)	(3.4)	(3.9)
- Coefficient on mediator (B_4_)	−2.4	−5.2*	2.5
	(2.6)	(3.0)	(3.5)
Service delivery disrupted due to broken equipment last 90 days			
- Coefficient on P4P (B_1_)	7.7***	3.9	11.4***
	(2.0; 13.3)	(−2.5; 10.3)	(3.7; 19.2)
- Coefficient on mediator (B_4_)	−5.0*	−5.3	−1.5
	(−10.6; 0.7)	(−11.7; 1.1)	(−9.2; 6.3)
Health worker knowledge			
- Coefficient on P4P (B_1_)	7.6**	4.3	10.1**
	(1.3; 13.9)	(−2.7; 11.3)	(1.7; 18.5)
- Coefficient on mediator (B_4_)	−7.2	−3.2	0.5
	(−17.9; 3.5)	(−15.2; 8.8)	(−13.8; 14.8)
Health worker kindness			
- Coefficient on P4P (B_1_)	7.8***	5.2	11.6***
	(2.2; 13.3)	(−1.1; 11.4)	(4.1; 19.0)
- Coefficient on mediator (B_4_)	0.3	−0.3	−0.11
	(−1.4; 2.0)	(−0.23; 0.16)	(−0.34; 0.12)
Proportion of women who paid for delivery in a HF			
- Coefficient on P4P (B_1_)	8.2***	3.0	9.9**
	(2.5; 13.8)	(−3.2; 9.1)	(2.4; 17.5)
- Coefficient on mediator (B_4_)	3.8	−27.7***	−16.6
	(−12.6; 20.2)	(−2.4; 1.7)	(−38.5; 5.2)

95% confidence intervals in parentheses; percent sign (%) indicates percentage points change; bold font indicates identified mediators

**p* < 0.10; ***p* < 0.05; ****p* < 0.01

**Table 8:** Indirect effect of potential mediators on maternal care outcomes

	Facility-based delivery (% change)	Public facility delivery (% change)
Effect of P4P without mediators		
Total effect	7.9***	7.4**
(Confidence Interval)	(2.4; 13.3)	(0.9; 13.9)
Indirect effect of P4P on outcome through mediators		
Oxytocin injection stock-out	3.9	5.0
Indirect effect (coefficient interaction)	0.024	0.030
Confidence interval	(−0.00; 0.138)	(−0.083; 0.143)
Percentage of total effect		
Health worker kindness		
Indirect effect (coefficient interaction)	0.1	
Confidence interval	(−0.019; 0.020)	
Percentage of total effect		
Percentage of women who paid for delivery in a HF		
Indirect effect (coefficient interaction)		2.3
Confidence interval		(0.4; 4.9)
Percentage of total effect		31.1

95% confidence intervals in parentheses; percent sign (%) indicates percentage points change; bold font indicates identified mediators *p < 0.10; **p < 0.05; ***p < 0.01

**Table 9:** P4P Average causal mediation effect (ACME) and direct effects, sensitivity analysis using Imai et al. (2010) approach

	Mediation analysis			Sensitivity results	
	Mean	[95% Conf.	Interval]		
Mediators for delivery in health facility					
Oxytocin stock-out					
ACME	0.017	0.006	0.028		
Direct effect	0.059	0.021	0.095	Correlation at which ACME = 0	−0.0439
Total effect	0.077	0.041	0.109		
% of tot eff mediated	0.226	0.159	0.424		
Health worker kindness					
ACME	0.039	0.031	0.048		
Direct effect	0.042	0.007	0.075	Correlation at which ACME = 0	0.2085
Total effect	0.081	0.045	0.116		
% of tot eff mediated	0.482	0.337	0.864		
Percentage of women who paid for delivery in HF					
ACME	0.041	0.032	0.051		
Direct effect	0.040	0.004	0.072	Correlation at which ACME = 0	−0.2326
Total effect	0.081	0.045	0.113		
% of tot eff mediated	0.511	0.364	0.911		
Mediators for delivery in public HF					
Oxytocin stock-out					
ACME	0.019	0.008	0.031		
Direct effect	0.037	0.000	0.077	Correlation at which ACME = 0	−0.0437
Total effect	0.057	0.021	0.096		
% of tot eff mediated	0.342	0.202	0.933		
Health worker kindness					
ACME	0.033	0.026	0.042		
Direct effect	0.031	−0.008	0.068	Correlation at which ACME = 0	0.161
Total effect	0.065	0.024	0.102		
% of tot eff mediated	0.516	0.325	1.374		
Percentage of women who paid for delivery in HF					
ACME	0.051	0.039	0.062		
Direct effect	0.014	−0.025	0.050	Correlation at which ACME = 0	−0.259
Total effect	0.064	0.025	0.100		
% of tot eff mediated	0.791	0.506	2.051		
Mediators for IPT2 uptake					
Health workers received supervision visit in last 90 days					
ACME	0.016	0.002	0.030		
Direct effect	0.102	0.038	0.161	Correlation at which ACME = 0	−0.0343
Total effect	0.117	0.056	0.174		
% of tot eff mediated	0.133	0.089	0.280		

*ACME* average causal mediation effect

## Abbreviations

ACME: Average Causal Mediation Effect
DiD: Difference in difference
LSEM: Linear structural equations model
P4P: Payment for performance
